# Phase-Amplitude Coupling in Spontaneous Mouse Behavior

**DOI:** 10.1371/journal.pone.0162262

**Published:** 2016-09-15

**Authors:** Daniel Thengone, Khatuna Gagnidze, Donald Pfaff, Alex Proekt

**Affiliations:** 1 Feil Family Brain and Mind Research Institute, Weill Cornell Graduate School of Medical Sciences, New York, New York, United States of America; 2 Laboratory of Neuroendocrinology, The Rockefeller University, New York, New York, United States of America; 3 Laboratory for Neurobiology and Behavior, The Rockefeller University, New York, New York, United States of America; 4 Department of Anesthesiology and Critical Care, University of Pennsylvania, Philadelphia, Pennsylvania, United States of America; University of Texas Southwestern Medical Center, UNITED STATES

## Abstract

The level of activity of many animals including humans rises and falls with a period of ~ 24 hours. The intrinsic biological oscillator that gives rise to this circadian oscillation is driven by a molecular feedback loop with an approximately 24 hour cycle period and is influenced by the environment, most notably the light:dark cycle. In addition to the circadian oscillations, behavior of many animals is influenced by multiple oscillations occurring at faster—ultradian—time scales. These ultradian oscillations are also thought to be driven by feedback loops. While many studies have focused on identifying such ultradian oscillations, less is known about how the ultradian behavioral oscillations interact with each other and with the circadian oscillation. Decoding the coupling among the various physiological oscillators may be important for understanding how they conspire together to regulate the normal activity levels, as well in disease states in which such rhythmic fluctuations in behavior may be disrupted. Here, we use a wavelet-based cross-frequency analysis to show that different oscillations identified in spontaneous mouse behavior are coupled such that the amplitude of oscillations occurring at higher frequencies are modulated by the phase of the slower oscillations. The patterns of these interactions are different among different individuals. Yet this variability is not random. Differences in the pattern of interactions are confined to a low dimensional subspace where different patterns of interactions form clusters. These clusters expose the differences among individuals—males and females are preferentially segregated into different clusters. These sex-specific features of spontaneous behavior were not apparent in the spectra. Thus, our methodology reveals novel aspects of the structure of spontaneous animal behavior that are not observable using conventional methodology.

## Introduction

Circadian rhythms are expressed in many behaviors and physiological functions including metabolism [1], and temperature regulation [2] in many animals [3] and in humans [4]. While circadian oscillations have been well known for many years, it has become clear that in addition to circadian oscillations many aspects of animal behavior and physiology fluctuate at faster (ultradian) time scales [5, 6]. Analysis of motor activity in several strains of rat has identified ultradian rhythms with cycle periods of 12 hr, 6 hr, 4.8 hr and 4 hr [7]. These ultradian oscillations may be influenced by both intrinsic factors and by the environment. For instance, ultradian oscillations in rabbits with cycle periods of 12.3 hr, 8.2 hr, and 6.1 hr [8] are influenced by light exposure [9]. One example of intrinsic regulation of such ultradian fluctuations is growth hormone secretion, which fluctuates with cycle period of .93 hr. [10, 11]. Furthermore, the neuronal basis of ultradian oscillations in mice [12, 13] entrained to 12h:12h light:dark cycle [14] has been shown to involve dopamine [15, 16]. Such co-existence of oscillations on multiple time scales raises an obvious question: Are different oscillations independent? If so, then the biological mechanisms that underlie different behavioral oscillations can be treated independently. Conversely, if different oscillations interact with each other then it would follow that the biological processes that give rise to different oscillations must be integrated. Here, we study fluctuations in spontaneous activity of mice and demonstrate that different behavioral oscillations are in fact strongly interdependent.

While traditional approaches to the analysis of periodicities in animal behavior that involve spectral estimation and autocorrelations are able to identify the frequency and power of different oscillators, they cannot provide insight into the interdependence between different oscillations. This is due to several strong assumptions about the data that are not obviously valid for animal behavior and physiology. The most critical assumption is that the statistical features of behavior (e.g. periodicity) remain constant. Yet even cursory examination of the behavior suggests that it is highly non-stationary. Previous reports have demonstrated that spontaneous behavior is characterized by bursts of activity punctuated by periods of rest across many species and environmental settings [17, 18]. Thus, distributions of many measures of activity are characterized by power-laws, long-range correlations and 1/f-like spectrum [19]. One important implication of this is that, there is no principled choice of a single time scale over which the behavior can be considered stationary and appropriately represented by its spectrum. Moreover, in traditional spectral analysis that relies on Fourier transform, the signal is convolved with a sine wave. To study oscillations at different frequencies the frequency of the sine wave can be adjusted. Yet regardless of the frequency, the sine wave extends indefinitely in time. This in effect embodies the assumption of stationarity in traditional spectral analysis. In wavelet analysis, the signal is convolved with a wavelet of a particular frequency. The major difference between wavelet and traditional spectral analysis is that the wavelet does not extend indefinitely in time [20]—increasing the wavelet frequency shrinks the wave in time while decreasing wavelet frequency leads to dilation of the wavelet. Thus, wavelet analysis allows one to study faster oscillations over shorter time scales and slower oscillations over longer time scales. Wavelet transform is thus ideally suited for the analysis of animal activity data since behavioral rhythms span a wide range of frequencies [21].

The major advantage of the wavelet transform is that it allows us to identify different oscillations in the behavior and study how the power (amplitude) of these oscillations changes over time. To anticipate the results, what we find is that the power of many oscillations fluctuates rhythmically, similar to AM (amplitude modulated) radio where the signal (slow oscillation) modulates the amplitude of a faster (carrier) oscillation. In other words, amplitude of one oscillation depends on the phase of a different, slower oscillation. We refer to this coupling between oscillators as phase-amplitude cross frequency coupling (CFC). In the context of biological systems, CFC has been mostly studied for a variety of brain-derived signals such as electroencephalogram (EEG), local field potential (LFP) and other brain recordings [22]. Much like animal behavior, spectra of many brain signals are 1/f-like. Yet the CFC analysis revealed that certain pairs of neuronal oscillators are tightly coupled while others are not, and that this coupling distinguishes scale-invariant brain activity from other scale-invariant signals such as seismic waves and stock market fluctuations [23]. Furthermore, the degree of coupling as well as the phase of coupling can be altered depending on the behavioral state [24]. In this study, we demonstrate that further analysis of the 1/f-like fluctuations in spontaneous behavior reveal additional structure not directly observable with previously applied analysis techniques.

## Results

### Wavelet analysis identifies ultradian behavioral oscillations

**Fig 1:**
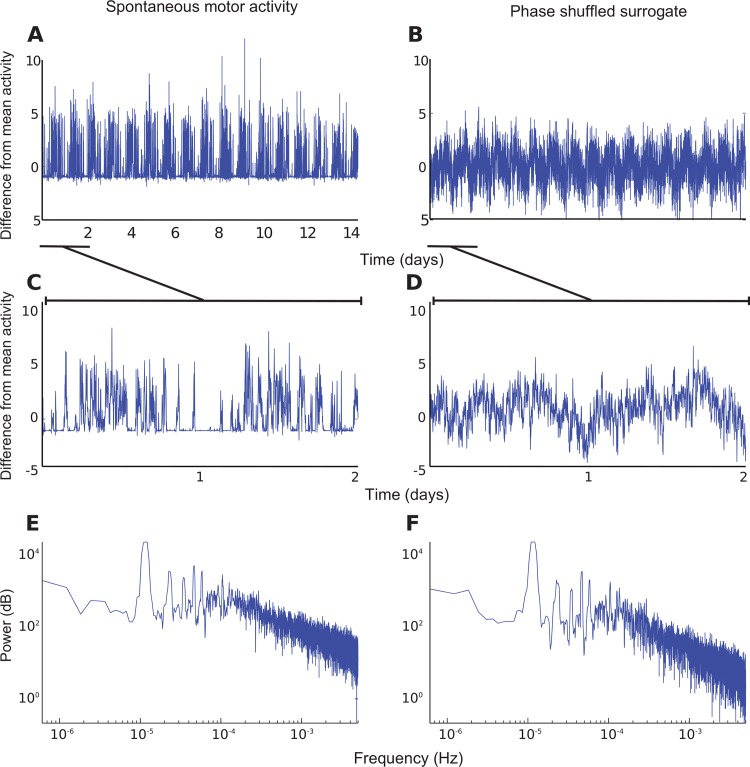
Spectrum does not capture the essential non-stationarities in spontaneous movements. **A.** Spontaneous motor activity in one male mouse over the course of a 14-day recording period (mean subtracted). An expansion of the trace in A over the period marked with a dashed line in shown in **C**. **B.** Phase-shuffled surrogate constructed on the basis of data in **A**. Expanded phase-shuffled surrogate trace is shown in **D**. Note that phase-shuffled surrogate is qualitatively different from the real data despite the fact that the power spectra of these data are the same (**E** real data, **F** phase-shuffled surrogate).

Fig 1A shows representative pattern of the locomotor activity during a 14-day recording session in one Het-8 male mouse. While it is clear that variations in the level of activity follow a cyclical pattern with the cycle period of ~ 24 hours, just specifying this periodicity (*i*.*e*. the spectrum) does not unequivocally capture the dynamics of spontaneous behavior. To illustrate this point, Fig 1B shows a phase shuffled surrogate of the data in Fig 1A (Methods). While the two signals have similar spectra (Fig 1E and 1F), in the time domain the real behavior is much more irregular and bursty than its surrogate (Fig 1C and 1D zooms in on the time axis for the real and the surrogate datasets respectively). The accentuated fluctuations reveal non-stationarity of animal behavior that is not captured by its power spectrum. In the remainder of the manuscript, we focus on these essential nonstationarities.

**Fig 2:**
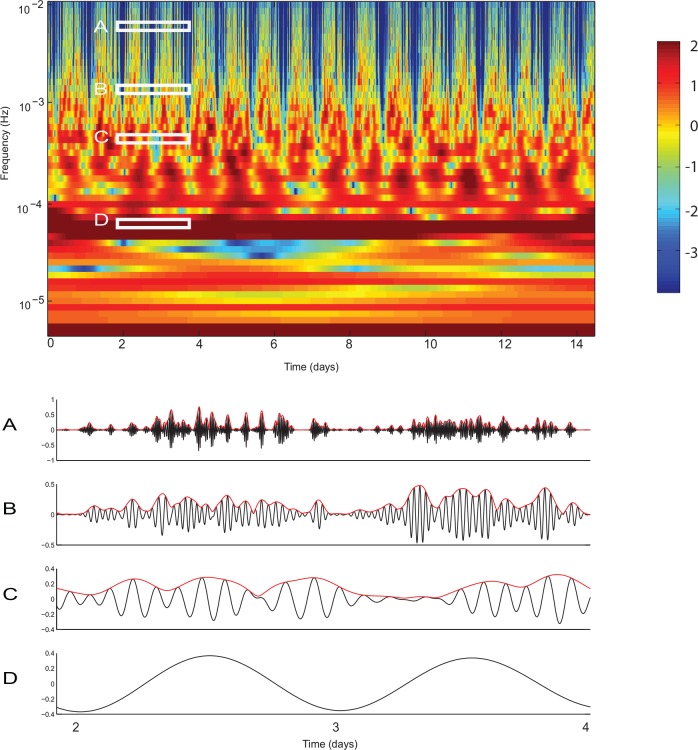
Continuous wavelet transform of locomotor activity reveals fluctuations in the power of multiple behavioral oscillations. Wavelet scalogram of spontaneous locomotor activity. Frequency is displayed on a logarithmic scale. Power of the oscillations is shown in color from blue (low power) to red (high power). The prominent horizontal ridge with high power corresponds to oscillations with frequency ~1/24 hours *i*.*e*. the dominant circadian oscillation. Multiple other oscillations are present, but the power of these oscillations fluctuates dramatically throughout the recordings. Continuous wavelet transform can be seen as a bank of filters centered at the frequency of each of the wavelets. **A-D** Data from the time-frequency regions indicated in wavelet spectrogram. **D** Corresponds to the circadian oscillation while **A-C** correspond to different ultradian oscillations. Black line in **A-D** shows the filtered locomotor activity, while red line shows the amplitude envelope of the oscillations computed as the absolute value of the Hilbert transform. While the amplitude of the circadian oscillation remains fairly constant in time, the amplitude of ultradian oscillations fluctuates.

In contrast to the power spectrum, continuous wavelet transform reveals the time-dependence of the amplitudes of oscillations at many different frequencies (Fig 2). Consistent with the observation of the data in the time domain, power at different frequencies fluctuates abruptly. To better appreciate these fluctuations, data filtered at 4 different frequencies (the regions in the time-frequency plane that correspond to each trace are shown with white rectangles) are shown beneath the wavelet scalogram. The amplitude of oscillations at many frequencies fluctuates in a rhythmic fashion (red lines in Fig 2A, 2B and 2C). This suggests that the amplitude of one behavioral oscillation may depend on the phase of another behavioral oscillation. In what follows we uncover this coupling by investigating the dependence of the amplitude of a faster oscillation on the phase of a slower oscillation.

### Illustration and intuition behind the analysis of phase amplitude coupling

**Fig 3:**
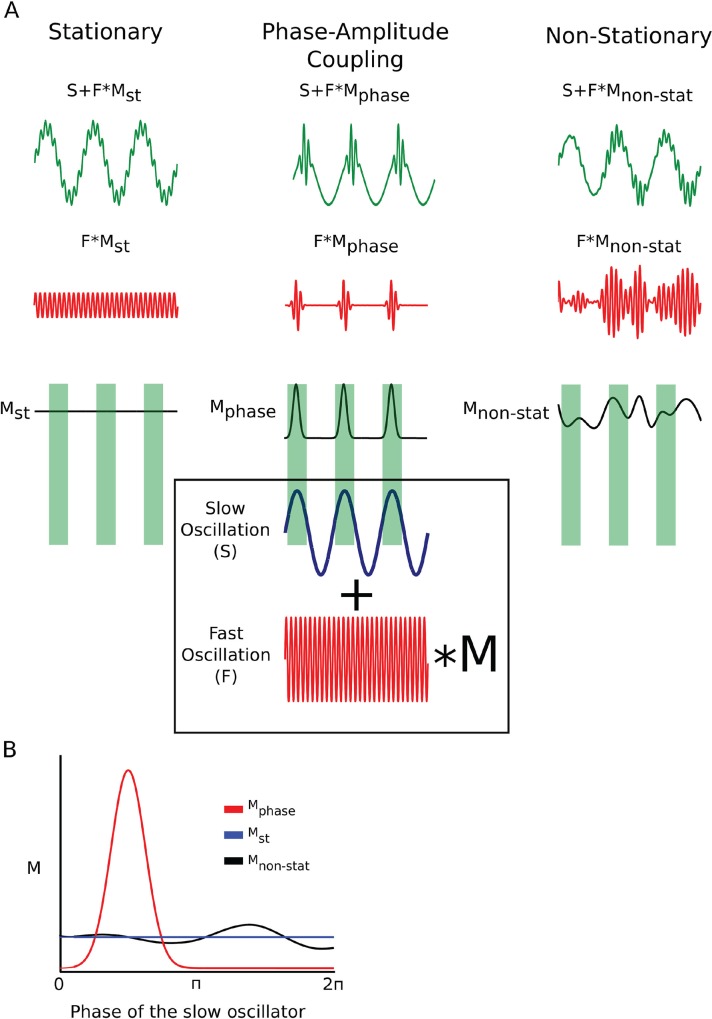
Illustration of phase amplitude modulation. **A.** Top row shows three time series. While they appear quite different in their characteristics, each of these time series is formed by adding two oscillations: fast (F) and slow(S) (highlighted by a box). The only difference between these three time series is that in each case the fast oscillation was multiplied by a function M (modulation). In the first case (left column), M_st_ is a constant function (left column, third row). Thus, the amplitude of the fast oscillation stays constant in time (left column, second row). The overall characteristics of the time series formed by addition of the fast and the modulated slow waves stays constant in time. Thus, this kind of pattern is called stationary. This is what is typically assumed in the analysis of behavioral oscillations. In the second column the fast oscillation is multiplied by M_phase_, a function that rises and falls depending on the phase of the slow oscillation. The relationship between M_phase_ and slow oscillation is highlighted by green rectangles. M_phase_ peaks at the peaks of the slow oscillation which corresponds to the phase of S = *π* / 2. For the purposes of illustration, M_phase_ was chosen to be a Gaussian distribution centered at *π* / 2. The net result is that the amplitude of the fast oscillation waxes and wanes rhythmically (second column, second row). Note the similarity between this example trace and real behavioral traces shown in Fig 2B and 2C. While phase amplitude coupling (second column) is an example of a non-stationary pattern, not all forms of non-stationarity imply phase-amplitude coupling. For example, consider the trace in the right column. The amplitude of the fast oscillation changes in time, but the waxing and waning of the fast oscillation does not have an obvious relationship to the phase of the slow oscillation (the lack of relationship is highlighted by the green rectangles). **B.** To determine whether phase of slow oscillator (S) modulates the amplitude of the fast oscillator (F), we re-plot the different modulating functions from **A** (M_st_, M_phase_, and M_non-stat_) as a function of phase of the slow oscillator rather than a function of time as in **A**. It can be seen that both M_st_ and M_non-stat_ do not change appreciably depending on the phase of the slow oscillator. In contrast, M_phase_ deviates significantly from uniform distribution. This deviation from uniform distribution is precisely what is measured by the modulation index, which quantifies the effect of phase of one oscillation on the amplitude of a different oscillation.

Fig 3A (top green traces) shows three example patterns. All three of them are constructed by linearly combining (adding) two sine waves: slow oscillation (S) and, fast oscillation (F) (highlighted by a rectangle). The only difference between the three top patterns is that prior to adding the two oscillations, the fast oscillation is multiplied by a different modulating function (M). In the first case (left), the modulating function is a constant (M_st_). Thus, the characteristics of the fast oscillation appear constant in time. This kind of pattern is called stationary and is adequately captured by the spectrum. In contrast, in the middle trace, the amplitude of the fast oscillation appears to grow dramatically at the peak of the slow oscillation (this corresponds to the phase of slow oscillation = *π* / 2). In this case the modulating function (M_phase_) approaches zero at all points except those near *π* / 2 of the slow oscillation. For the purposes of illustration, M_phase_ was chosen to be a Gaussian distribution centered at *π* / 2 of the slow oscillation. The correspondence between the phase of the slow oscillation and the peaks in M_phase_ are highlighted by green rectangles. Multiplication of the fast oscillation by M_phase_ gives rise to the waxing and waning of the fast oscillation (second row, middle column) depending on the phase of the slower oscillation. This is an example of phase-amplitude cross frequency coupling (CFC). Note, however, that not all non-stationarities imply CFC. In the rightmost trace (top), the amplitude of the fast oscillation changes in time (i.e. the signal is non-stationary). Yet the waxing and waning of the fast oscillation is not connected in an obvious way to the phase of the slower oscillation. This is illustrated by showing that M_non-stat_ does not appear to depend on the phase of the slower oscillation. The objective of the analysis that follows is to first extract the oscillations occurring at different frequencies (akin to F and S in the example). This step is accomplished by filtering the behavioral time series using wavelets centered at different frequencies (e.g. Fig 2). The second step is to quantify the amplitude of the fast oscillation (akin to M in the example) and the phase of the slow oscillation. This is accomplished using a Hilbert transform (Methods). Amplitude extracted from filtered behavioral time series is illustrated by red traces in Fig 2. The final step is to determine whether amplitude of the fast oscillation depends on the phase of a slower oscillation. In order to accomplish this last step we simply plot amplitude of one oscillation as a function of phase of a different, slower oscillation. We illustrate this for the cartoon examples in Fig 3 by plotting M_st_, M_phase_, and M_non-stat_ as a function of phase of S. It can be readily seen that only M_phase_ depends strongly on the phase of S. This implies that in this particular case the two oscillations are coupled.

### Modulogram analysis reveals phase—amplitude coupling in spontaneous behavior

To extract oscillations occurring at frequencies *f*_*i*_ and *f*_*j*_, we filtered the data using wavelets centered at *f*_*i*_ and *f*_*j*_ (where *f*_*j*_ > *f*_*i*_ and *f*_*i*_ ≥ 1/24 *hours*). To quantify the interactions between these oscillations, we first computed the distribution of amplitudes of *j (A*_*j*_*)* using Hilbert transform. We then plotted *A*_*j*_ (after normalization as described in the methods) as a function of phase of *i* (Φ_*i*_). As illustrated in Fig 3, if *A*_*j*_ is independent of Φ_*i*_ then the plot of *A*_*i*_ vs Φ_*j*_ is expected to be a flat line. Thus, deviation from the flat line can be used as a measure of dependence of *A*_*j*_ on Φ_*i*_. This was quantified using Kullback-Leibler (KL) divergence (Methods).

**Fig 4:**
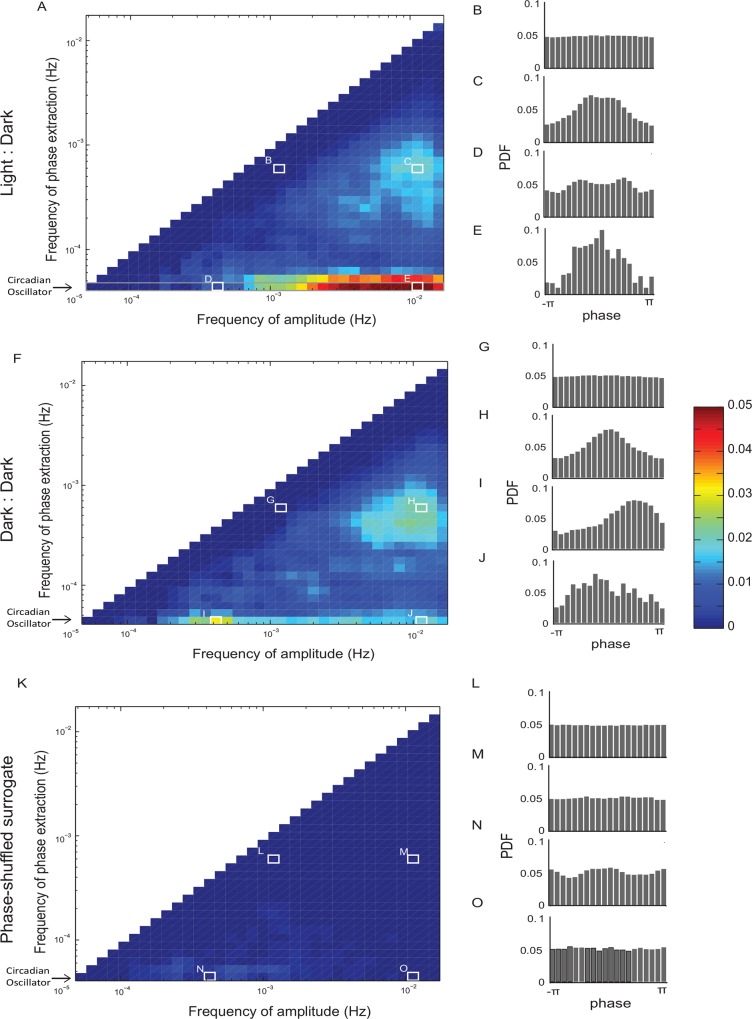
Multiple behavioral oscillations are coupled. The frequency of the oscillator used for amplitude extraction (the modulated wave) is plotted on the abscissa in modulograms (**A, F, K**). The frequency of the oscillator used for phase extraction (modulator) is plotted on the ordinate. Modulation Index (MI) is plotted in color from blue (no modulation) to red (significant modulation) on the same scale for all modulograms. Examples of the phase vs. amplitude histograms from the regions in the modulogram (white rectangles) are shown in panels (**B:E**, **G:J**, **L:O**). **A.** Modulogram showing strengths of cross frequency phase-amplitude coupling in spontaneous behavior observed during light:dark cycle. The prominent horizontal line along the x-axis with high modulation indices corresponds to the multiple ultradian oscillators modulated by the circadian oscillator. Some oscillators are being modulated by the ultradian oscillations (e.g. **C**). Some oscillations appear to be uncoupled (e.g. **B**). Finally some oscillations are modulated by more than one oscillator (e.g. **E** and **C**). **F.** Modulogram in the same animal maintained in total darkness. While some modulation is clearly present and thus reflects the intrinsic properties of spontaneous behavior, the overall pattern of modulation depends quite strongly on the lighting conditions. Note the decreasing influence of the circadian oscillator and an increasing influence of some ultradian oscillations. **K.** In contrast to the experimentally-observed datasets, the phase shuffled surrogates computed from the same data as in **A** (or **F**, not shown) do not display significant phase-amplitude coupling despite having identical spectra.

We refer to the normalized value of the KL divergence as modulation index (MI) following the approach [25] (Methods). We compute the MI for all pairs of oscillations (Fig 4). While circadian oscillation is clearly dominant and modulates several faster oscillations (bottom row of the modulograms shown in Fig 4A, 4F and 4K, example of *A*_*j*_ vs Φ_*i*_ plot for a particular ultradian oscillator that is modulated by the circadian oscillation is shown in Fig 4E), other oscillations can also contribute to modulation. Some faster behavioral oscillations are modulated by more than one slow oscillation (e.g. Fig 4C and 4E). Yet other pairs of oscillations appear to be largely uncoupled (Fig 4B). To assess the statistical significance of the observed MI values, we compared them to those observed in the phase shuffled datasets (Fig 4K). For each real dataset, we constructed 500 phase-shuffled surrogates and computed the distribution of MI values for each frequency pair. Then each experimentally observed MI can be expressed in terms of its probability under the assumption of null hypothesis given by phase shuffling. Using this procedure, statistically significant coupling was observed in every animal (*n = 59* mice) both under 12h:12h light-dark conditions (Fig 4A, 4B, 4C, 4D and 4E) as well as in animals maintained in total darkness (Fig 4F, 4G, 4H, 4I and 4J). Note that some modulation indices that were prominent during light:dark conditions became less prominent in constant darkness (e.g. Fig 4E and 4J) while others became more prominent (Fig 4D and 4I). Presence of statistically significant coupling between different oscillations observed in total darkness reflects variables intrinsic to each animal rather than those imposed onto the animal extrinsically by the light/dark cycle. Thus, MI analysis reveals novel and statistically robust elements of the temporal structure of spontaneous behavior that is not given by the spectrum.

### Identifying characteristic features among groups of animals

We observed that while there were consistent features of the map of the CFCs, different individuals taken from genetically diverse populations of mice exhibited some variability in the specific features of these interactions. Furthermore, the same animals maintained under light:dark (Fig 4A) and in constant darkness (Fig 4F) conditions exhibited different patterns of CFCs. One possibility for the observed differences is that they simply reflect noise inherent in both the estimation of the CFC and in the behavior itself. Alternatively, differences in the pattern of CFC may reflect systematic differences between animal phenotypes and environmental influences such as the light:dark cycle. While in principle the pattern of CFC can reflect some combination of intrinsic properties of the animal and features of the environment, the complete investigation of the variability in the patterns of CFC would require a large number of animals and an efficient parameterization of the environmental factors. Thus, here we focused just on the variability in the patterns of CFC computed in the simplest setting–constant darkness and in one generation of *Het8* mouse strain. To study the variability in the pattern of CFC, we first subjected the CFC maps (e.g. Fig 4) to dimensionality reduction using principal component analysis (PCA), and then clustered the data in this reduced dimensional space.

**Fig 5:**
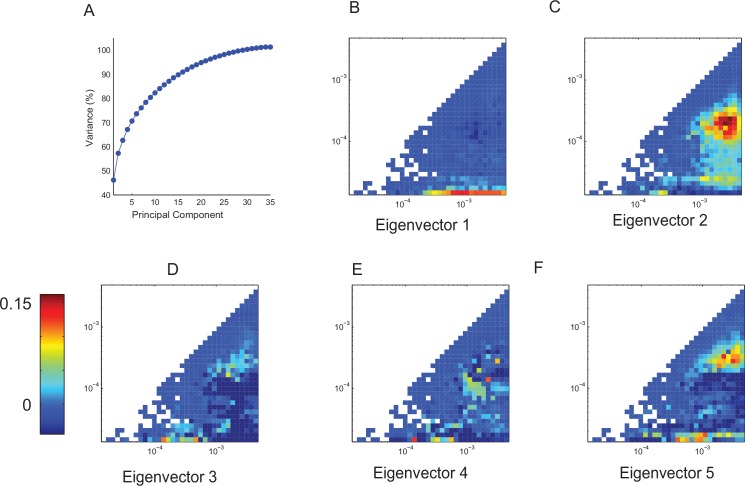
Differences in the modulograms computed for different animals under constant darkness are confined to a low dimensional subspace. **A.** Activity from 35 individual *Het8* mice (19 females, 16 males) were observed in total darkness for 14 days. The modulograms computed for these data were subjected to principal component analysis. The percent of total variance is plotted as a function of the number of principal components (dimensions). While there is no distinct elbow in this plot, first 5 components capture >70% of the variance. **B-F**. Principal components (eigenvectors of the correlation matrix) represent the characteristic patterns of CFC. While the first eigenvector extracts the influence of the circadian oscillator, subsequent eigenvectors pick out the interactions between different ultradian oscillators.

Using PCA, we captured ~70% of the variance in the first 5 components (Fig 5A). Thus, variability between different individuals in terms of patterns of CFC is confined to a low dimensional subspace. Formally, the principal components (PCs) that span this low dimensional space are the eigenvectors of the covariance matrix computed across all pairs of CFC maps and reflect characteristic and mutually orthogonal patterns of CFCs (Fig 5B–5F). As expected, the first PC captures the effect of the most dominant modulator–the circadian oscillation (Fig 5B). Subsequent components, however, highlight the interactions between different ultradian oscillations (Fig 5C, 5D, 5E and 5F).

**Fig 6:**
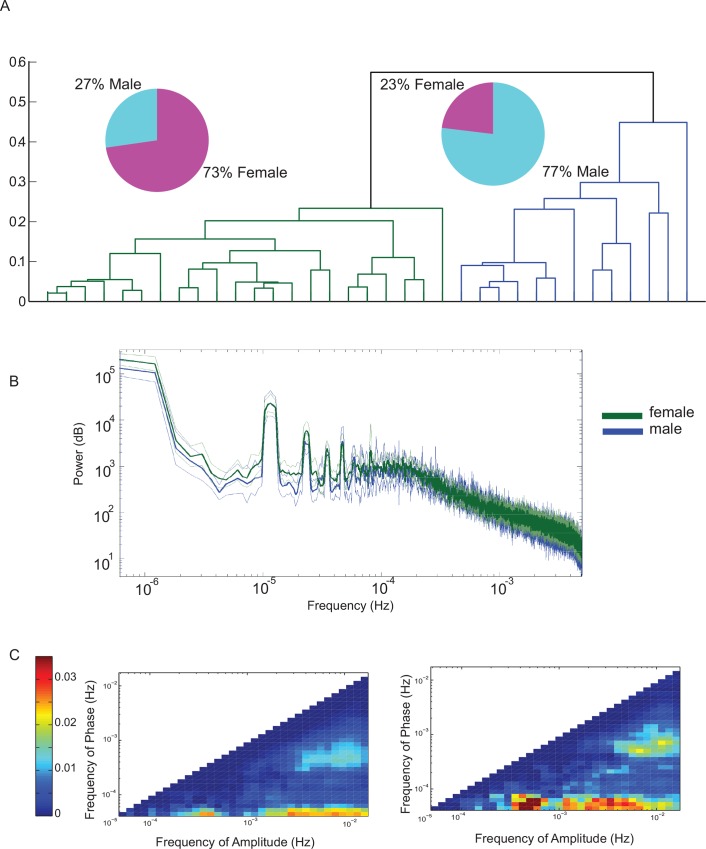
Males and females can be segregated into two clusters based on the CFC but not on the spectra. **A:** Dendrogram computed for the same animals as in Fig 4 reveals that much of the variability can be captured by 2 clusters. Euclidean distance between elements is shown on the *y-axis*. Clustering was done on the CFC maps projected onto the first 5 PCs. One cluster contains data predominantly from female (73%) while the other is enriched in the male mice (77%) (shown by the pie charts). The probability of obtaining this degree of segregation between males in females under null hypothesis of random assignment is *p* = 0.00635 (Fisher exact probability). **B:** In contrast to the separation between males in females on the basis of the CFC, the spectra from the two populations show no clear differences. **C:** Centroids of the two clusters (left→ female enriched cluster; right→ male enriched cluster) reveal clear differences in terms the strength of coupling between different ultradian oscillators.

To discover further structure in the differences between individual patterns of CFCs, we subjected the data projected onto the first 5 PCs to cluster analysis using hierarchical clustering (Fig 6A, Fig 6C shows centroids of each of the clusters). The dendrogram reveals that there are two clusters of CFCs. While one cluster is enriched in the females, the other cluster predominantly contains males and this degree of segregation between different sexes is statistically significant (*p =* 0.00635, Fisher exact probability). In contrast to the CFC, the spectra obtained from males and females were quite similar (Fig 6B). Thus, analysis of the CFC reveals novel features of spontaneous locomotor activity differentially expressed in males and females.

## Discussion

In this study, we find that ultradian rhythms in spontaneous behavior are coupled to the circadian rhythm and to each other. Conventionally, spontaneous behavior has been analyzed using spectral or autocorrelation analysis—techniques that are not appropriate to reveal the patterns of interactions among the different oscillators due to the stationarity assumption. By using wavelet transform we relaxed this assumption to discover highly significant interactions between phases and amplitudes of behavioral oscillations at different frequencies. To demonstrate statistical significance, we compared the pattern of interactions observed in the real data to that computed over phase-shuffled surrogates constructed to preserve the spectral characteristics of the signal.

Circadian oscillations are mediated through a feedback regulatory network of transcription factors. Since the original groundbreaking discovery of the period mutants by Konopka and Benzer [26] the number of genes thought to be important for mediating cyclical fluctuations in animal behavior and physiology has increased tremendously [27]. Along with the discovery of the new genes, the network defined by their interactions has also gained in complexity. It seems likely that such complex molecular network has evolved to not simply give rise to the circadian oscillation but to coordinate the various rhythms of behavior and physiology across broad range of timescales. Yet, the methods currently applied to the analysis of behavior are not appropriate to detect such coordination between different behavioral rhythms. Analysis of phase amplitude coupling between different behavioral oscillations developed herein is a step towards a more complete characterization of behavior. Indeed our analysis suggests that coupling between different behavioral rhythms occurs in the absence of any external clues. This implies that the coupling is a consequence of the intrinsic properties of the molecular network that underlies different biological rhythms.

While the intrinsic components of the circadian oscillations are important, previous studies have demonstrated that the specific features of the circadian oscillator depend critically on the environment [28]. We find that this is also true of the cross frequency coupling between different behavioral oscillations. While coupling is observed both in the light:dark cycle and in constant darkness [29, 30], the specific pattern of coupling in these two settings is distinct. (Fig 4A–4J). Furthermore, we observe that the pattern of CFC can differ significantly between different individuals from an outbred mouse strain. Interestingly, this variability is not entirely random [31, 32] implying that only certain configurations of CFC are most biologically plausible.

We show that just by analyzing the patterns of CFC in a featureless dark environment we can identify features that are more characteristic of either males or females. While it is tempting to speculate on the biological significance of the sex differences in CFC, segregation of the sexes into two clusters is not entirely surprising. Sex differences were the only consistent biological difference between the animals included in the analysis. Further analysis will be required to pinpoint the relationship between the patterns of CFCs and sex. The most important aspect of the clustering result is that the pattern of CFC depends on the underlying biological differences between animals—sex in this case—rather than being some generic feature of the 1/f-like fluctuations in the amount of locomotion. This segregation of males and females into different clusters, while statistically significant, was not perfect. In this context, we note that the presence of scalloping activity in the behavioral rhythms in female hamsters (and in mice to a lesser degree) has been linked to their natural estrous cycle (approximately 4 days) [33, 34, 35]. However those findings and the ones herein are distinct—the most important distinction being timescale. While work on scalloping has addressed the interactions between the circadian rhythm and the hormonal oscillation occurring at a slower time scale, here we exclusively focused on oscillations with the cycle period that is at most circadian. While it is possible that some of the differences in coupling revealed by the CFC may reflect estrous cycle, it is not possible to make any specific claims without disrupting the estrous cycle experimentally. We emphasize, however, that our objective here was not to come up with a classification scheme to distinguish between the sexes. This would likely be possible given larger datasets and more robust classifiers. What our results do suggest quite strongly is that the differences between males and females were not observed using conventional methods of analysis of spontaneous behavior.

Not surprisingly, our analysis of the CFC identified circadian oscillation as the key, but not the only, modulator of faster ultradian oscillations. Mutations in the Clock gene have been shown to lengthen the circadian cycle with notable shifts in the circadian spectral peaks [36], and deficiency in Bmal1 gene completely disrupts the circadian rhythm [37] as evidenced by the disappearance of the spectral peak at the circadian frequency. Yet, it is not obvious what effects, if any, this should have on the modulation of the ultradian oscillations. For instance, assume that the slow oscillation (S) in Fig 3A is the circadian oscillation, while the fast oscillation (F) is an ultradian oscillation. Elimination of the circadian oscillation without the concomitant elimination of the modulation will result in the transformation of the behavioral time series from the green trace (Fig 3A, top row, middle column) into the red trace (Fig 3A, second row, middle column). Note that the presence of modulation in this case will not show up as a spectral peak at the circadian frequency consistent with the results in Bmal1-deficient animals. Conversely elimination of both the circadian oscillation and the modulation will result in either a stable ultradian oscillation (Fig 3A, second row, left column) or a non-stationary ultradian oscillation (Fig 3A, second row, right column). Furthermore, the converse scenario—elimination of the modulation without elimination of the periodicity—is also possible. In other words, the modulation demonstrated herein is a fundamentally distinct phenomenon from the oscillation itself.

While little is known about the mechanisms of ultradian oscillations, recent study by Blum et al, 2015 [15], suggests that dopaminergic neurons may be strongly involved. This is evidenced for instance by pharmacological manipulation of dopaminergic signaling using psychostimulants and antipsychotics as well as genetic manipulations of the dopaminergic signaling. Of note, however, Blum et al, were able to demonstrate most profound alterations in the cycle period of the ultradian oscillations after disrupting the circadian oscillation. This is because it is thought that the pronounced and relatively stable circadian oscillation “masks” the ultradian oscillations that are by comparison much more transient. The approach laid out in this work may therefore be useful both in the characterization of the basic features of the ultradian oscillations such as their cycle period and amplitude but also in their integration into the overall coordinated patterns of behavior that involves oscillations occurring over a broad range of frequencies.

## Conclusion

Spontaneous behaviors, like many biological features and physiological mechanisms are complex processes that are highly non-stationary—their statistical character fluctuates at slow and fast timescales as a result of interactions with a fluctuating environment. To account for these dynamical patterns, it is essential to reduce the signal into its components and elucidate the underlying interactions between them. The wavelet based coupling analysis of the components presented here identified a highly non-random phase structure in the spontaneous behavior that was relatively preserved across all mice. Moreover, studying the alterations in locomotor dynamics would potentially be useful for behavioral abnormalities such as those observed in psychiatric disorders like bipolar disorder and depression [38].

More generally, we note that the methodology employed here could be applied to spontaneous human behaviors in a variety of settings. Analysis of the CFC applied to behavior on a larger scale, may pinpoint both the idiosyncrasies of individuals and the organization of these individuals into subgroups.

## Methods

### Animal Maintenance and Data Acquisition

All animal procedures were approved by The Rockefeller University's Animal Care and Use Committee in accordance with the Animal Welfare Act and the Department of Health and Human Services Guide for the Care and Use of Laboratory Animals. Food and water were available ad libitum. Mice were housed separately in acrylic cages (18cm x 29cm x 13cm). Two strains of mice, C57BL/6J (Jackson Labs) and Het-8 (an outbred stock from an intercross of more than eight outbred strains followed by over 60 generations of structured outbreeding, were maintained on either 12h:12h light:dark cycle or in constant darkness. For both strains adult animals between 3 and 6 months of age were used. Only recordings longer than 14 days were used in this study. Animals were housed individually inside a VersaMax monitor placed within a chamber, and maintained at a constant temperature of 20 (±2°C and the food and water were provided *ad libitum*. The procedure for data acquisition is specified in detail in [19]. Briefly, locomotor activity measured using breaks of IR beams arranged in a grid in a Versamax monitor enclosed in a chamber is used to assay mouse spontaneous behavior. The cages do not impose any restriction in the direction of movement within the boundaries of the box or any timing cues. Each individual Versamax apparatus was housed inside a sound-proof chamber each with its own dedicated ventilation system in order to limit the possibility of interactions between individuals. The experiments were performed in a dedicated room and all attempts were made to limit the number of entries into the room during the experiment. Thus, to the best of our ability we minimized any external time cues. To reduce the computational cost for the purposes of the analysis here we reduced the temporal resolution to 1 sample per 100 seconds by down-sampling the data (see the analysis section below).

### Data Analysis

All analysis was performed in Matlab (Mathworks) using custom-written software. While the original data sets were acquired at 1 second temporal resolution for computational feasibility we downsampled the data using Matlab function *decimate*.*m* to 100 seconds (0.01 Hz). All subsequent analysis was performed on this downsampled time series.

Power spectrum of the behavioral timeseries was computed using multitaper estimates [39]. This method provides for robust, unbiased spectral estimation even given a relatively short period of observations. Here we used 5 tapers and a time-bandwidth product of 9.

### Construction of surrogates

For each experiment, we established a statistical control analysis to infer whether the observed coupling, quantified with the MI value (see below), differed from a chance distribution derived from the analysis of surrogate data. We constructed two sets of surrogates designed to preserve the spectral features while altering the phase information. The downsampled data was first transformed from time to frequency domain using the Fourier transform. Phase information was extracted and randomized in one of two ways (below), and transformed back into the time domain using the inverse Fourier transform. By construction these surrogate datasets have the same power spectrum as the original timeseries. Thus, all first order correlations are preserved in the experimental timeseries (Wiener-Khinchin theorem).

Two procedures were used to create 500 surrogate time series for each experimental dataset: 1) Phases are sampled randomly from a uniform phase distribution between −*π* and *π*. We refer to this as phase randomized surrogates. Note that this not only randomizes the phases but also disrupts the phase distribution. 2) The phases of the original data were shuffled randomly between the different frequency components. Note that this, second method, preserves both the power spectrum and the distribution of phases found in the original dataset while disrupting only the relationship between phase and frequency. We refer to these as phase shuffled surrogates. While all of the conclusions in the paper hold for both sets of surrogates, here we focused on the phase-shuffled surrogates because they preserve more of the features of the real data than the phase-randomized dataset.

### Continuous Wavelet Transform (CWT)

CWT of a signal x(t) is defined as the convolution of the signal with a scaled and translocated basis function known as the mother wavelet, Morlet in this case. The choice of the mother function is neither unique nor arbitrary. The requirements open the possibility of using several different functions as wavelets [20]. We chose the Morlet wavelet because the resulting CWT is complex-valued, thus allowing us to extract both phase and amplitude information. Moreover, the presence of sinusoidal characteristics of the time series supports the use of this wavelet function.

The CWT of a signal *x*(*t*) is defined in [40]:

$$X(b,a)=-\sqrt{\left|a\right|}\int_{-\infty }^{\infty }x(t)\varphi ((t-b))/a)dt$$ (Eq.1)

where the Morlet wavelet *φ*(*t*) is defined by the following equation:

$$\varphi (t)=\left(e^{iw_{0}t}-e^{\frac{w_{0}^{2}}{2}}\right)e^{\frac{t^{2}}{2}}$$ (Eq. 2)

in which *w*_0_ = 6 is the so called wavenumber parameter chosen to assure that admissibility criteria for the wavelet are met [20].

*a* in (Eq 1) is called wavelet scale. The relationship between wavelet scale and frequency is given by the Fourier transform of the wavelet ([20], Table 1). Changing the scale (*a)* has the effect of shifting the peak of the Fourier transform of the Morlet wavelet and changing the width of the peak in the frequency domain. In the time domain changing *a* has the effect of broadening or shrinking the wavelet. This is the essence of the wavelet transform—different time windows are used to estimate the characteristics of oscillations occurring at different frequencies. Please see excellent guide to wavelet analysis by Torrence et al 1998 [20] for the details of the implementation and the interpretation of the wavelet transform.

Using standard approach we performed the wavelet transform along a dyadic scale grid where each octave (doubling of the frequency) was divided into 4 elements. Given the data sampling rate (*dt* = 100 s or 0.01 Hz), the smallest wavelet scale (*a*_0_) that can be computed is given by *a*_0_ = 2 * *dt (Nyquist limit). The largest scale is $a_{\max}=a_{0}2^{j*d_{j}}$ where d*_*j*_ *is the spacing between consecutive scales (1/4 in this case) $j=\frac{\log_{2}(N*\frac{dt}{a_{0}})}{d_{j}}$ where N* is the number of points in the original data series. We only considered datasets that were at least 2 weeks in duration. This yields a minimum number of points 12095 (where each point denotes activity within a 100 s period).

While the actual mechanics of the wavelet transform are done in terms of scales, it is much more convenient and conventional to report the results in terms of actual frequencies (where each *a* is associated with a frequency that corresponds to the peak of the Fourier transform of the Morlet wavelet). We refer to each frequency in the manuscript by *f* and, following SI units report frequency in terms of Hz. Each particular frequency element within a set of all estimated frequency elements is denoted by a subscript (e.g. *f*_*i*_ *corresponds to the i*^*th*^ *frequency element)*.

While CWT is very useful for time-frequency decomposition of the signal (see Fig 2), another way to see the CWT is as a bank of bandpass filters. We used this property to generate multiple filtered versions of the behavioral dataset, one at each of the wavelet scales. This was accomplished by performing an inverse CWT using just the wavelet of interest. This procedure avoids phase shifts commonly encountered with filtering because wavelet coefficients at each scale are computed independently.

### Analysis of Phase-Amplitude Coupling

The approach for calculation of phase-amplitude coupling is identical to that discussed in [25]. Below we outline this strategy. Please consult Tort et al [25] for the discussion of this measure of phase amplitude coupling as well as other alternative measures.

To establish whether the phase of oscillation occurring at frequency *f*_*i*_ modulates the amplitude of the oscillation occurring at frequency *f*_*j*_ the behavioral time series *x*(*t*) is first filtered using wavelets with scales chosen such that the peak of the frequency response of the wavelet is at *f*_*i*_ *and f*_*j*_. Phase of signal filtered at *f*_*i*_ is then extracted using standard Hilbert transform. This yields phase time series (denoted as Φ_*i*_(*t*)). The instantaneous amplitude of the oscillation filtered at frequency *f*_*j*_ was similarly extracted by taking the absolute value of the Hilbert transform. This yields amplitude time series (denoted as *A*_*j*_(*t*)). Composite phase-amplitude time series [*φ*_*i*_(*t*),*A*_*j*_(*t*)] is then constructed. Note that the phase and amplitude are extracted from different oscillations distinct in terms of their frequency. Then the phase (Φ_*i*_(*t*)) is binned into 20 bins on the range (−*π*,*π*) and mean amplitude of *A*_*j*_(*t*) in each bin is computed. We denote this mean amplitude in k^th^ bin as ⟨*A*_*j*_⟩_*k*_. Note that this assumes that the relationship between phase Φ_*i*_(*t*) and amplitude *A*_*j*_(*t*) is stationary across the recording. While this not need be the case necessarily, in order to test this assumption, longer recordings would be necessary. Finally mean amplitudes in each bin are normalized according to the following $P(k)=\frac{{\langle A_{j}\rangle }_{k}}{\sum_{r=1}^{N}{\langle A_{j}\rangle }_{r}}$ where N = 20 (i.e. number of bins). This normalized amplitude has properties similar to probability density function in that *P*(*k*) ≥ 0∀*k* and $\sum_{k=1}^{N}P(k)=1$.

Note, that the intensity of the phase-amplitude coupling can be inferred visually by inspecting the plot of *P*(*k*) (e.g. Fig 3 and Fig 4). If Φ_*i*_(*t*) and *A*_*j*_(*t*) are not related (i.e. no coupling) then the plot will appear as a flat line or a uniform distribution. Conversely, if the phase modulates amplitude, then the normalized amplitude will not be uniform across phases.

Thus, in order to quantify the phase-amplitude coupling we compute the deviation of *P*(*k*) from uniform distribution (*U*(*k*)). For this purpose we invoke Kullback-Liebler (KL) divergence, a standard approach to compute differences between distributions in statistics and information theory. For two discrete distributions (*P* and *U* in this case) KL divergence is defined by the following equation:

$$D_{KL}(P,U)=\sum_{k=1}^{N}P(k)\log\left(\frac{P(k)}{U(k)}\right)$$ Eq. 3

Note the resemblance between *Eq 3*and equation for Shannon entropy $H(p)=-\sum_{k=1}^{N}P(k)\log(P(k))$. Further note that the maximum entropy value occurs at *log(N)*. Finally, note that maximum entropy occurs precisely when the distribution is uniform. Thus, we can rewrite Eq 3 as follows: *D*_*KL*_(*P*,*U*) = log(*N*) − *H*(*P*). Because *H*(*P*) ≤ log(*N*) we defined our modulation index for pair of frequencies *f*_*i*_ and *f*_*j*_ (identically to [25]) as follows:

$$MI_{i,j}=\frac{D_{KL}(P,U)}{\log(N)}$$ Eq. 4

Note that if *P*(*k*) is a uniform distribution then the numerator of *Eq 4*is zero. Thus, if two oscillations are not coupled, MI is zero. Conversely, maximum coupling is seen when *P*(*k*) is a Dirac delta function (non-zero amplitude for oscillation with frequency *f*_*j*_ is only observed in a single phase bin of oscillation with frequency *f*_*i*_). In this case, MI will attain its maximum value (MI = 1).

We performed the above analysis for all pairs of frequencies sampled according to the procedure described in the previous section. For the purposes of plotting the modulation index is shown in color in corresponding figures. *f*_*i*_ and *f*_*j*_ are shown on the ordinate and the abscissa respectively. The frequency pairs were chosen such that 1/24 hours ≤ *f*_*i*_ < *f*_*j*_. That is, we only considered oscillations that are at most as slow as the circadian oscillation and only allowed for the possibility that slower oscillation modulates a faster one but not vice versa. The former requirement can in principle be relaxed but would require longer contiguous recordings. The latter requirement gives rise to the triangular appearance of the modulograms.

In order to establish, statistical significance of the MI, we applied the same procedure to phase-shuffled and phase-randomized surrogate datasets (see above). 500 surrogates were used for each animal. The MI values derived from the original data and the surrogate datasets were compared to obtain a Z-score measurement of cross-frequency phase-amplitude coupling strength. In every experiment on every mouse we found robust statistically significant (*p<0*.*0001* after Boneferroni correction for multiple comparisons*)* coupling between different behavioral oscillations.

## Supporting Information

S1 Dataset The attached matlab structure contains the activity data for the animals used in this study. The fields labeled with “DD” corresponds to the animals raised in the total darkness, and those without the “DD’ label corresponds to the animals raised in 12h:12h light:dark cycle. The activity is recorded for 14–17 days, and the gender of the animals is denoted by F (females) or M (males) respectively
(ZIP)
